# Supplementary material for: A Global Screen for Assembly State Changes of the Mitotic Proteome by SEC-SWATH-MS
Source: Cell Syst. 2020 Feb 26;10(2):133–155.e6. doi: 10.1016/j.cels.2020.01.001 (PMC7042714; doi:10.1016/j.cels.2020.01.001)

Q99873 | ANM1_HUMAN | PRMT1 HMT2 HRMT1L2 IR1B4
Monomer MW [kDa]: 41.516 Monomer expected elution fraction: 48

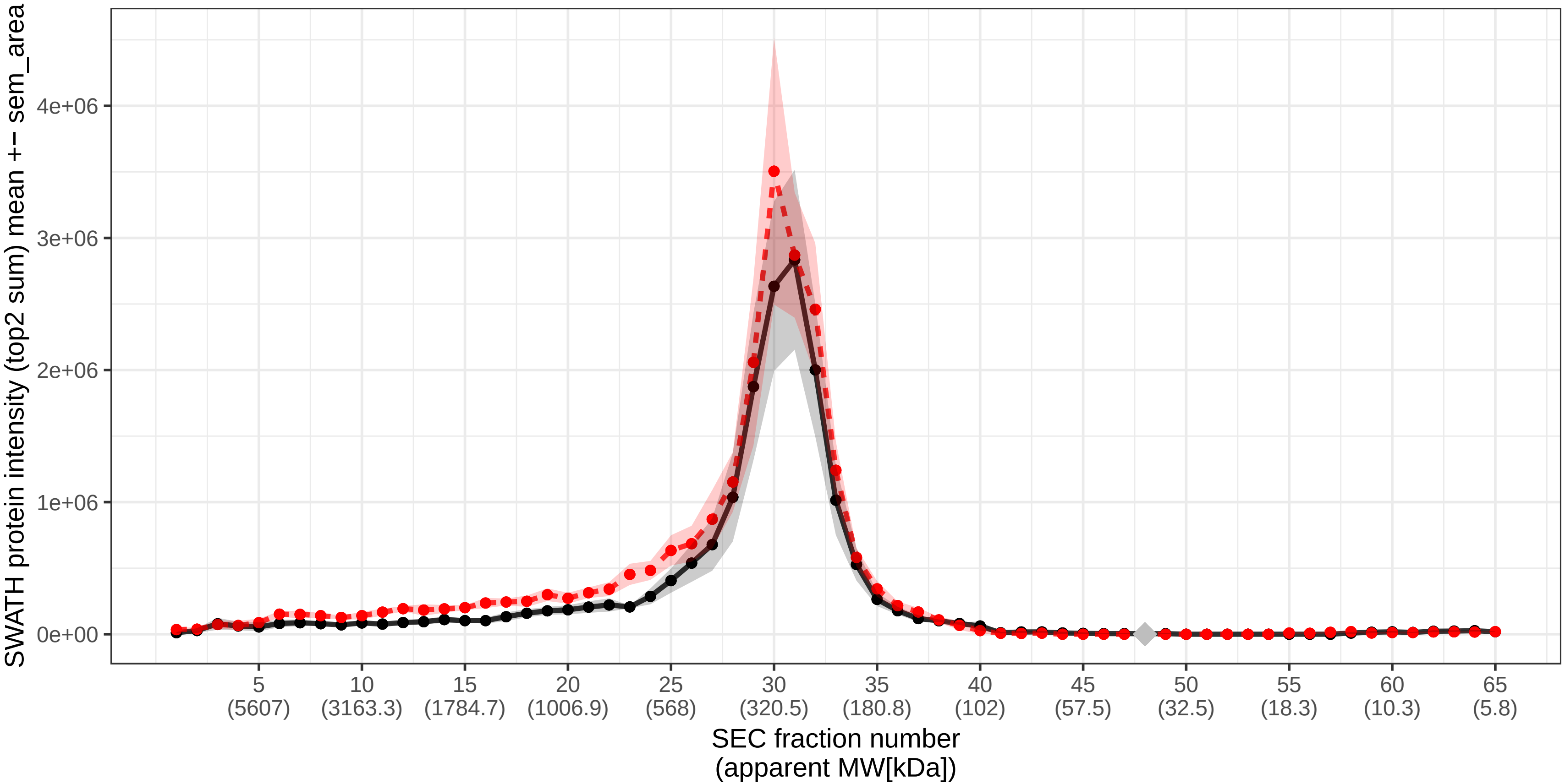

Supplement: Data S1. SEC-SWATH-MS Protein Chromatograms, Related to Figure 1 [file mmc6.zip › SECchrom_Q99873_ANM1_HUMAN_PRMT1_HMT2_HRMT1L2_IR1B4.pdf]
